# A Co-Polarization Broadband Radar Absorber for RCS Reduction

**DOI:** 10.3390/ma11091668

**Published:** 2018-09-09

**Authors:** Thtreswar Beeharry, Riad Yahiaoui, Kamardine Selemani, Habiba Hafdallah Ouslimani

**Affiliations:** 1 Laboratoire Energétique Mécanique Electromagnétisme, Université Paris Nanterre, 92410 Ville d’Avray, France; ryahiaoui@u-paris10.fr (R.Y.); habiba.ouslimani@parisnanterre.fr (H.H.O.); 2 Constructions Mécanique de Normandie, Systems department, 50105 Cherbourg, France; kselemani@cmn-cherbourg.com

**Keywords:** absorber, broadband, single layer, rcs reduction

## Abstract

In this article, a single layer co-polarization broadband radar absorber is presented. Under normal incidence, it achieves at least 90% of absorption from 5.6 GHz to 9.1 GHz for both Transverse Electric (TE) and Transverse Magnetic (TM) polarizations. Our contribution and the challenge of this work is to achieve broadband absorption using a very thin single layer dielectric and it is achieved by rotating the resonating element by 45°. An original optimized Underlined U shape has been developed for the resonating element which provides a broadband co-polarization absorption. The structure is 12.7 times thinner than the wavelength at the center frequency. To understand the absorption mechanism, the transmission line model of an absorber and the three near unity absorption peaks at 5.87 GHz, 7.16 GHz and 8.82 GHz have been used to study the electric and magnetic fields. The physical insight of how the three near unity absorption peaks are achieved has also been discussed. After fabricating the structure, the measurements were found to be in good agreement with the simulation results. Furthermore, with the proposed original UUSR resonating element, the operational bandwidth to thickness ratio of 6.43 is obtained making the proposed UUSR very competitive.

## 1. Introduction

Since the first successful experimental work on Metamaterials (MMs) reported by Smith et al. [1], various metamaterial-based applications have been developed covering low to optical frequencies [2,3,4,5,6,7,8]. MMs are no longer only a research field; they have now in fact proven to be successful in industrial solutions. MMs have been deployed for several applications such as compact antennas [9], invisible cloaking [10], super lenses [11] and sensing [12] to name but a few. MMs are also useful candidates for electromagnetic wave absorbers [13,14,15]. Nowadays, absorbers are required for Electro-Magnetic Compability (EMC) and stealth (radar cross section) applications. Broadband capability in defense and EMC applications of MMs is limited by their resonant structures providing narrow bandwidth. Extensive research on narrow band [16,17], multiband [18,19] and large band [20,21,22] Metamaterial Absorbers (MAs) have been published. To enlarge the bandwidth, two of the most used techniques consist of incorporating resonating elements working at nearby frequencies arranged on the same plane [23] or stacked on several layers [24]. Both techniques lead to a drop in the absorption (sometimes the reflection coefficient S11 > −10 dB) at certain frequencies in the bandwidth. Using several elements on the same plane is very difficult to implement due to lack of space in a small unit cell. On the other hand, multiple layers can considerably increase the total height of the structure which is not desired for many applications. Using well customized magnetic materials can decrease the height [25] but they can be very expensive. Using active components [26,27] such as diodes can improve bandwidth and absorption but some defense applications require the least possible external electronic sources. Some recent works [28,29] have shown that broadband absorption using simple shapes of resonating elements can be achieved but the use of materials such as Titanium, Tin Oxide or complicated fabrication processes makes the absorbers more costly. Replacing the metallic resonating elements by well designed resistive sheets [30] can provide broadband absorption but depending on the values (Ω/*Square*) it is sometimes very difficult to manufacture them due to limited technology. For these reasons, thin broadband MAs designed with easily available dielectrics especially for low frequencies and the gigahertz regime are very complicated to design and are a challenging topic. In this paper, we present a thin broadband MA of 3.5 GHz of bandwidth operating in the band of 5.6 GHz to 9.1 GHz achieving more than 90% of co-polarization absorption in the whole band at normal incidence. The co-polarization absorption rate remains more than 50% until 40° of oblique incidence for both Transverse Electric (TE) and Transverse Magnetic (TM) polarizations. As it will be seen in the Design section, the resonator (only the metallic Underlined U shape) is rotated by 45° and due this rotation the cross-polar absorption is not significant. Depending on applications, not achieving cross-polar absorption is more or less critical. Inside radomes of military vessels for example, cross-polar absorption is not often required for the reduction of electromagnetic interference.

## 2. Results

### 2.1. Design of the Unit Cell

**Figure 1:**
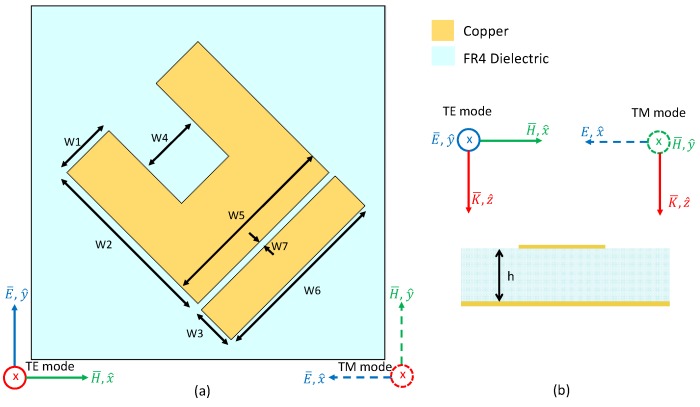
(**a**) Top view of the unit cell of the proposed absorber, (**b**) Cross sectional view of the uniet cell.

The unit cell of the absorber is illustrated in Figure 1. It consists of a metallic Underlined U Shaped Resonator (UUSR) deposited on a metal backed FR4 dielectric. The UUSR is axially rotated by 45°. The FR4 dielectric is 3.2 mm thick and is backed with metal to prevent transmission. A 0.017 mm thin copper having electric conductivity of 5.8 × 10^7^ S/m was used for the UUSR and to back the FR4. The FR4 has a permittivity of 3.92 and loss tangent of 0.025. The unit cells are arranged in periods of P = 15 mm. Numerical design and simulations were performed using the commercial software CST Design Studio Suite 2018. Periodic boundary conditions were applied in the numerical model in order to mimic a 2D infinite structure. The dimensions in milliliters (mm) are as follows. W1 = 2.5, W2 = 7.85, W3 = 1.8, W4 = 2.85, W5 = 7.85, W6 = 8, W7 = 0.175, L = 4.35, P = 15, h = 3.2. The USSR is centered at (x = −0.112611, y = 0.112611).

### 2.2. Simulation Results

**Figure 2:**
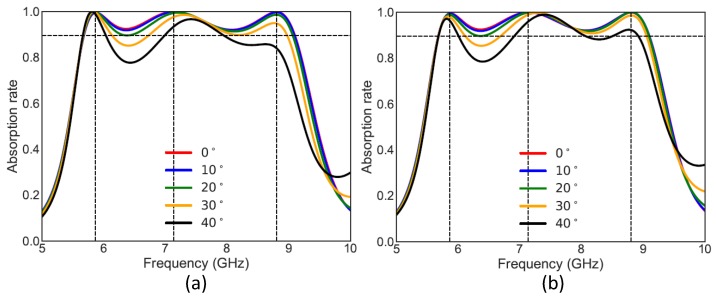
Absorption rate for normal and oblique incidences for (**a**) linearly polarized TE wave and (**b**) linearly polarized TM wave.

The co-polarization absorption rate for normal and oblique incidences of linearly polarized TE and TM waves of our structure is presented in Figure 2. The figure indicates that, for both TE and TM polarizations, for normal incidence and oblique incidences until 20°, the absorption rate is more than 90% in the whole range of 5.6–9.1 GHz frequency band. The absorption rate is more than 85% and 77% for 30° and 40° respectively. Three near unity absorption peaks have been observed at 5.86 GHz, 7.16 GHz and 8.82 GHz and are be used to understand the absorption mechanism in the next chapter.

**Figure 3:**
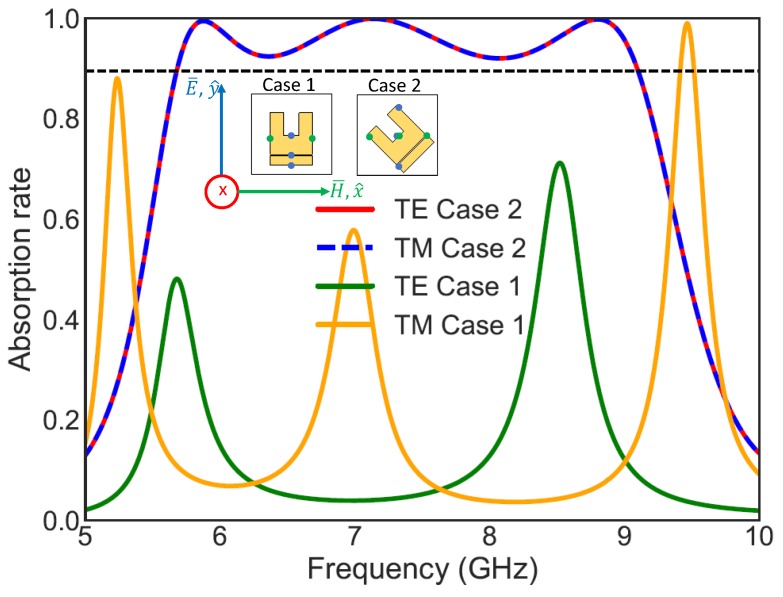
Absorption rates for linearly polarized TE and TM waves for Case 1 and Case 2.

As described in the introduction, the broadband absorption is obtained by rotating the UUSR by 45°. This strategy has been adopted in some recent works such as [24]. To understand how broadband absorption is obtained, the absorption results for both TE and TM linearly polarized waves of the absorber is presented for two cases in Figure 3. The first case (Case 1) represents the unrotated UUSR and the second case (Case 2) represents the UUSR rotated by 45°. The UUSR has been inspired from U shapes which exhibit capacitive response due to gap between the two arms. The whole shape was then optimized using CST Studio.

For the first case (Case 1), when the UUSR is not rotated the segments of the patch (UUSR) excited by the electric field (blue points on the patch) is not the same as the segments excited by the magnetic field (green points on the patch). Hence, the absorption rates for TE represented by the green curve and TM represented by orange curve are not the same. Moreover, broadband absorption is not achieved. Therefore, the patch oriented as in case 1 is not efficient. When the UUSR is rotated by 45°, as in Case 2, the structure becomes more interesting as the electrical and magnetic field interact with exactly the same segments of the patch. TE and TM modes become equal as shown by red and blue curves. Furthermore, the absorption is considerably increased and broadened.

### 2.3. Absorption Mechanism

**Figure 4:**
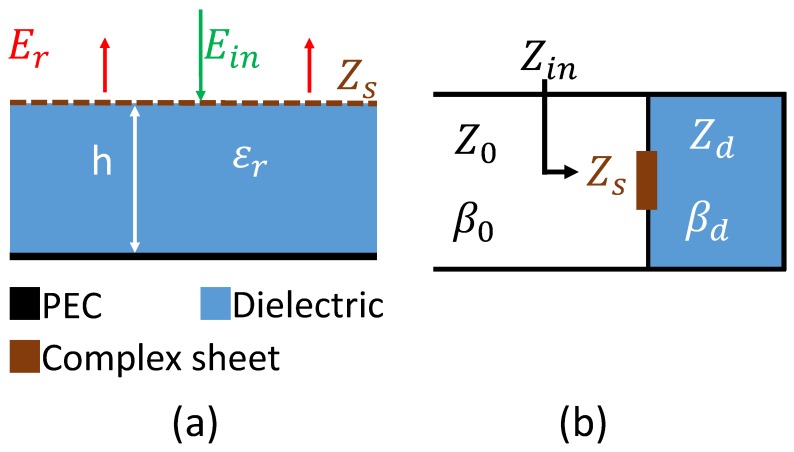
(**a**) Schematic design of a radar absorber, (**b**) Transmission line model of the absorber.

To understand the absorption mechanism the transmission line model of an absorber can be considered. One straight forward solution to design a radar absorber is to place a complex sheet (surface providing complex impedance) over a grounded dielectric as shown in Figure 4. The complex sheet is designed in a way that the whole structure matches the impedance of the free space ($Z_{0}$ = 377 Ω). Three parameters are important for the design of the absorber: the thickness and permittivity of the dielectric substrate and the geometrical parameters of the complex sheet.

Using the transmission line theory, the impedance of the grounded substrate for normal incidence is given by [31]:

(1) $$Z_{g}=jZ_{d}tan\left(\beta_{d}h\right),$$

where $Z_{d}=\sqrt{\mu_{0}/\epsilon_{0}\epsilon_{r}}$ is the characteristic impedance of the dielectric and $\beta_{d}$ = $\frac{\omega \sqrt{\epsilon_{r}}}{c}$ is the propagation constant in the dielectric. The input impedance, $Z_{in}$, is given by the parallel combination of the grounded dielectric ($Z_{g}$) and the complex sheet ($Z_{s}$):

(2) $$Z_{in}=\frac{jZ_{s}Z_{d}tan\left(\beta_{d}h\right)}{Z_{s}+jZ_{d}tan\left(\beta_{d}h\right)}$$

For perfect absorption, the reflexion must satisfy *R* = $\left(Z_{in}-Z_{0}\right)/\left(Z_{in}+Z_{0}\right)$ = 0. Hence, perfect absorption is obtained when $Z_{in}$ = $Z_{0}$. The required impedance of the complex sheet, $Z_{req}$, for perfect absorption, satisfying $Z_{in}$ = $Z_{0}$ is given by:

(3) $$Z_{req}=\frac{Z_{0}Z_{d}tan\left(\beta_{d}h\right)}{jZ_{0}+Z_{d}tan\left(\beta_{d}h\right)}$$

**Figure 5:**
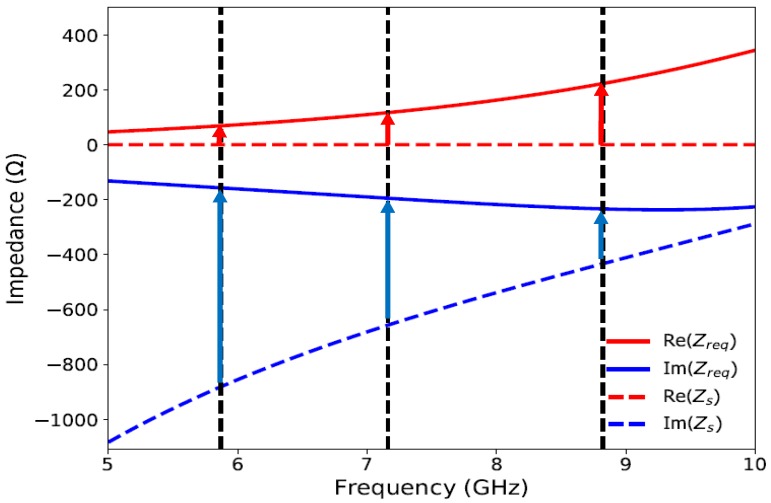
Required real (Re($Z_{req}$)) and imaginary (Im($Z_{req}$)) parts for perfect absorption and real (Re($Z_{s}$)) and imaginary (Im($Z_{s}$)) parts of the impedance of the UUSR.

From Equation (3), it can be seen that the required complex sheet for perfect absorption depends on the permittivity and the thickness of the dielectric. The required complex impedance ($Z_{req}$) of a 3.2 mm thick FR4 dielectric substrate is plotted according to Equation (3) in Figure 5 in the range of 5–10 GHz and are compared to the complex surface impedance of the UUSR ($Z_{s}$). $Z_{s}$ is the complex surface impedance of the UUSR without the dielectric and ground plane.

**Figure 6:**
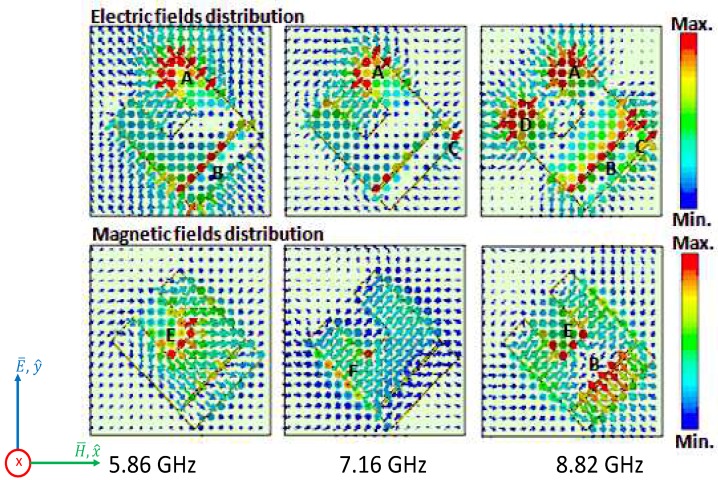
Distribution of electric and magnetic fields on the absorber at 5.86 GHz, 7.16 GHz and 8.82 GHz for a linearly polarized TE wave. Different labeled regions correspond to regions where maximum electric or magnetic fields have been observed.

It can be observed from Figure 5 that the real part of impedance of the UUSR (Re($Z_{s}$)) is very close to zero which is logical as the UUSR is made of copper. It should be noted that positive values of the imaginary parts mean an inductive coupling is taking place and negative values mean that a capacitive coupling is taking place. In our case, Re($Z_{s}$) being very close to zero and Im($Z_{s}$) being negative, the surface impedance $Z_{s}$ is capacitive and can be written in the form $Z_{s}$ = 1/jCω. *C* is the value of the equivalent capacitance. For a good absorption (if not perfect), Re($Z_{s}$) and Im($Z_{s}$) must be as close as possible to Re($Z_{req}$) and Im($Z_{req}$) respectively. For broadband absorption, firstly, an inductive coupling is required such that Im($Z_{s}$) increases and becomes close to Im($Z_{req}$) and secondly losses must be introduced such that Re($Z_{s}$) increases and becomes close to Re($Z_{req}$). Placing the UUSR on top of the metal backed lossy FR4 dielectric substrate will increase both Re($Z_{s}$) and Im($Z_{s}$). The real part of the surface impedance, Re($Z_{s}$) will be increased as the dielectric substrate is lossy and the inductive coupling to increase Im($Z_{s}$) will be produced by anti parallel currents between the UUSR and the ground plane provided that the dielectric is very thin compared to the wavelength. Obviously, electric coupling is also induced in the structure. The figure below depicts the electric and magnetic fields at the three near unity absorption peaks (Figure 6).

**Figure 7:**
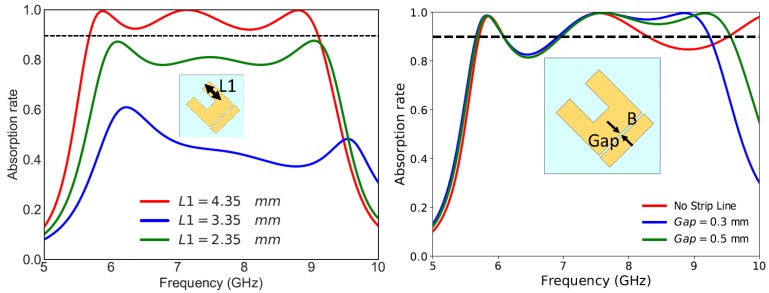
(**a**) Absorption rate for different values of L1, (**b**) Absorption rate for different values of the gap. An illumination of linearly polarized TE wave is considered.

For the first near-unity absorption frequency at 5.86 GHz, the electric fields are mainly produced around regions A and B. The magnetic fields are caused by the anti-parallel currents (not shown in the paper) flowing at regions E. At 7.16 GHz, the electric fields are created by regions A and C. The magnetic fields are mainly provoked by the anti-parallel currents flowing at region F and the ground plane. For the final near unity absorption peak at 8.82 GHz, the electric fields are mainly produced around regions A, B, C and D. The magnetic fields are introduced by the anti-parallel currents flowing at regions E, B and the ground plane and also by the anti parallel currents flowing between the bottom of the UUSR shape and the top of the strip line. The coupling of electric and magnetic fields led to near unity absorption. As we can observe for the three frequencies, the electric fields are, among others, confined around region A (right hand of the UUSR). Decreasing the length, L1, of the right hand of USSR should decrease the absorption in the whole band. We can check this by tuning the length, L1, of the right hand of the UUSR. It was observed that the capacitance increases and while L1 decreases. Thus the absorption rate is deteriorated when L1 decreases as shown in Figure 7a.The magnetic fields around region B contributed to the third absorption peak only. No magnetic fields were found around region B for the first and second absorption peaks. Thus, modifying the distance between the strip line and the U shape will affect only the third absorption peak as shown in Figure 7b.

**Figure 8:**
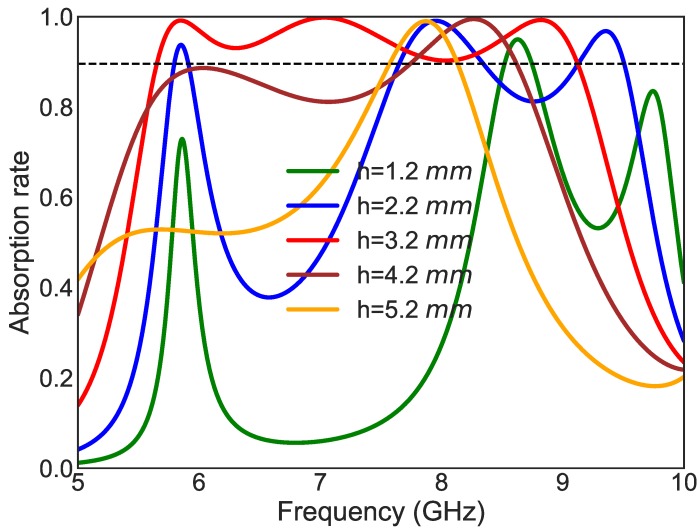
Absorption rate for different values of thickness (h), An illumination of linearly polarized TE wave is considered.

The selection of dielectric thickness is a very important parameter in the design of a broadband absorber. Increasing the thickness of an absorber does not necessarily increase the absorption bandwidth. What is more important is that the surface the parallel combination of the UUSR impedance and the grounded dielectric impedance matches the impedance of air. Moreover different thicknesses will provide different type of coupling. Figure 8 depicts the absorption rate for different thicknesses. An illumination of linearly polarized TE wave is considered.

### 2.4. Measurements and Verifications

**Figure 9:**
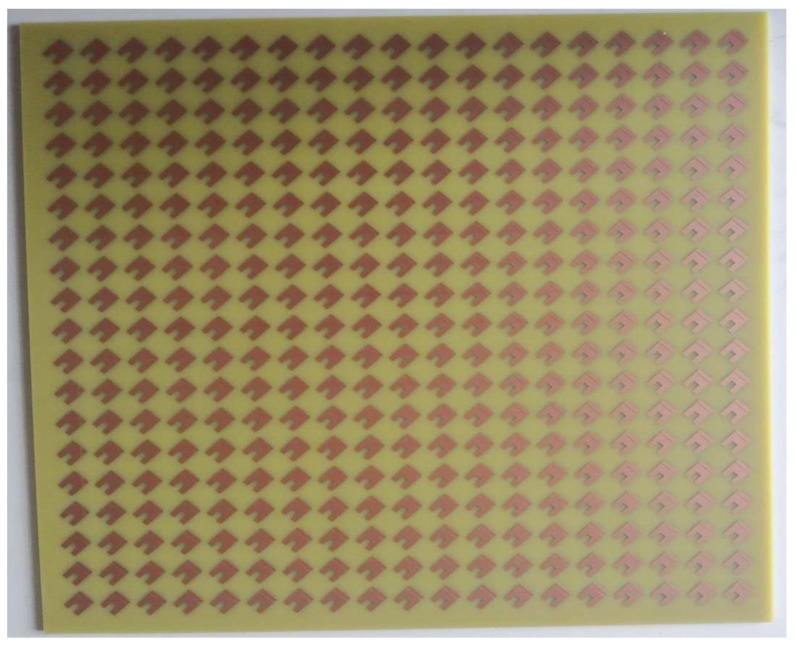
The fabricated 300 mm × 300 mm prototype.

An experimental prototype with dimensions of 300 mm × 300 mm and consisting of 19 × 19 unit cells was fabricated using conventional printing circuit board (PCB) technology (as shown in Figure 9).

**Figure 10:**
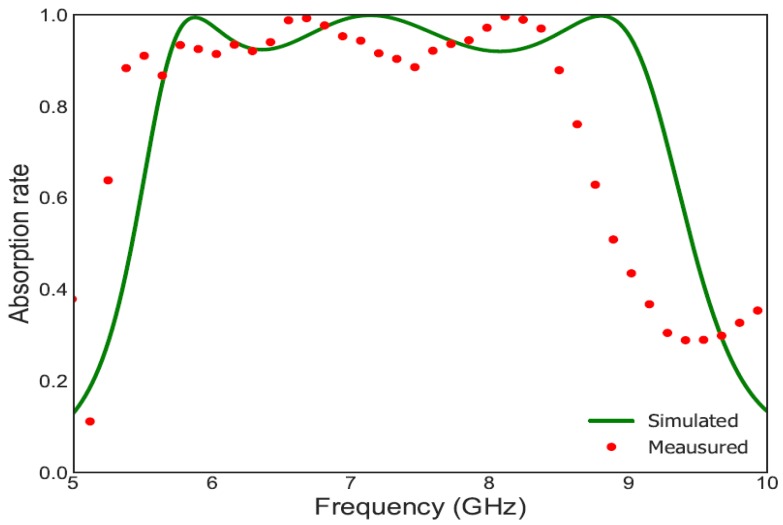
In red circles, the measured absorption rate is plotted and in green the simulated absorption rate is shown. Both are plotted under normal incidence for linearly polarized TE waves.

For a linearly polarized TE wave the simulated (solid green line) and measured (red circles) amplitudes of the absorption rate under normal incidence are plotted in Figure 10. Good agreement in terms of bandwidth and absorption rate is reported between simulation and measurement. For the measured absorption rate, the slight shift of the whole frequency band towards lower frequencies can be explained by the fact that the exact value of the permittivity of the fabricated FR4 dielectric. Simulations have shown that a small variation of the value of FR4 (for example 4 instead of 3.92) shifts the whole frequency band towards lower frequencies.

## 3. Discussion

The performance of an absorber is often judged upon its 90% absorption rate band to center frequency ratio given by $\left(f_{max}-f_{min}\right)/f_{c}$, where $f_{c}$ is the center frequency. This criteria does not take into consideration the thickness of the absorber which can be the most important factor in some applications. The operational bandwidth to thickness ratio to evaluate the performance of the absorber is used this work. Operational bandwidth to thickness ratio is given by $(\lambda_{fmin}-\lambda_{fmax})/h$, where $\lambda_{fmin}$ and $\lambda_{fmax}$ are the wavelengths at $f_{min}$ and $f_{max}$ respectively and h is the thickness of the absorber. The bigger is this ratio, the better operational bandwidth to thickness ratio performance an absorber has. Table 1 is a comparison of designed absorbers in this paper with some other absorbers.

**Figure 11:**
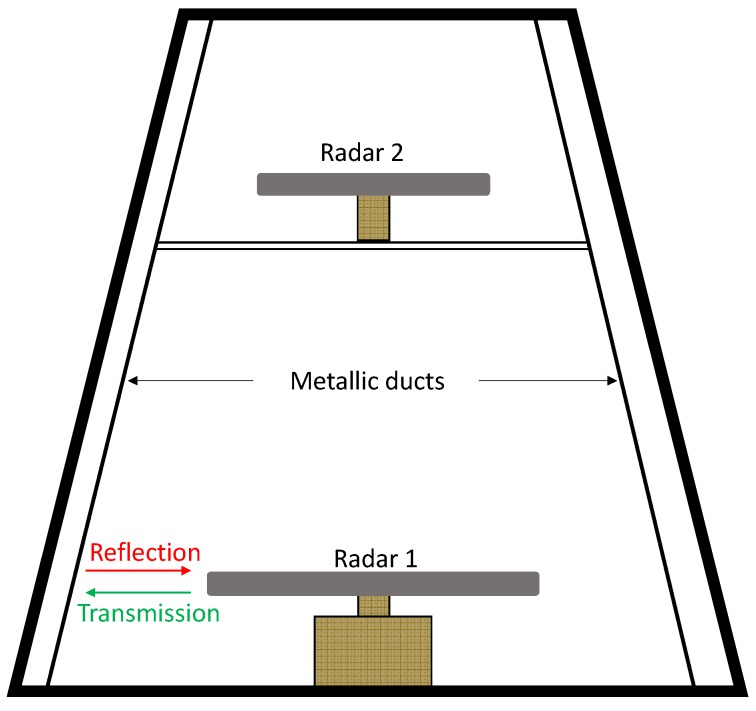
Representation of the profile view of radars mounted inside a radome.

Even if the proposed design is valid for co-polarization, it can be clearly seen from Table 1 that, a very high performance can be achieved with the proposed resonating element (UUSR). In fact, an operational bandwidth to thickness ratio of 6.43 is obtained making the proposed UUSR very competitive. Further work can be done to enlarge the bandwidth by optimizing multi layers. Communication devices such as detection radars working on the X band frequency range, one suitable application of the absorber is to reduce back scattering from metallic objects in radomes of military vessels. For example, several antennas and radars are mounted inside radomes which may contain metallic objects such as cable ducts. Reflected electromagnetic waves due to these metallic objects cause EMI critical issues such as saturation of source antennas and radars. The metallic ducts also cause indirect echoes, shadow and blind zones. Covering metallic parts with radar absorbers can decrease considerably the reflections, and hence the indirect echoes. This is illustrated in Figure 11 .

## 4. Materials and Methods

### 4.1. Simulation

Numerical designs and simulations were performed using the commercial software CST Design Studio Suite 2018. Periodic boundary conditions were applied in the numerical model in order to mimic a 2D infinite structure. Floquet ports were used for the excitation of the periodic structure. Simulation results were plotted using a free mathematical programming language tool.

### 4.2. Measurement

Measurements have been done in an anechoic chamber using a vector network analyzer. Two broadband FLANNR^®^ horn antennas working in the 2–18 GHz frequency band are used as emitter and receiver in reflection configuration. The reflection coefficient is normalized using a sheet of copper as reflecting mirror.

## 5. Conclusions

A single layer low profile broadband co-polarization absorber exhibiting more than 90% of absorption in the whole band of 5.6 GHz to 9.1 GHz. The proposed structure consists of an Underlined U Shape Resonator deposited on a dielectric. We have discussed the absorption mechanism by studying the transmission line model and the magnetic fields. A prototype of the absorber was fabricated and measurement results are in good agreement with numerical results. Due to the proposed original UUSR resonating element, the operational bandwidth to thickness ratio of 6.43 is obtained making the proposed UUSR very competitive and useful for future works. One very important limitation of the proposed absorber is that the absorber is not suitable cross polarization absorption due to the shape of the UUSR. As discussed in the introduction some applications require only co-polarization absorption. Future works can be done in order to make the absorber flexible for non planar objects. This can be done by using flexible dielectrics such as rubber and by optimizing the UUSR. Also further work of optimization must be done in order to use it for non planar objects as the boundary conditions used in this work are for flat targets.

## Figures and Tables

**Table 1:** Performance comparison of our absorber with some other absorbers.

Reference	90% Absorption Bandwidth (GHz)	Thickness (mm)	(*λ_fmin_* − *λ_fmax_*)/*h*
[32]	7–18	4.36	6
[33]	5.8–12.2	5	5.42
[34]	8.37–21	3.65	5.9
[35]	40–134	1	5.26
This work	5.6–9.1	3.2	6.43
